# Polygenic Risk Scores in Alzheimer's Disease: Current Applications and Future Directions

**DOI:** 10.3389/fdgth.2020.00014

**Published:** 2020-08-11

**Authors:** Emily Baker, Valentina Escott-Price

**Affiliations:** ^1^UK Dementia Research Institute at Cardiff University, School of Medicine, Cardiff University, Cardiff, United Kingdom; ^2^MRC Centre for Neuropsychiatric Genetics and Genomics, Division of Psychological Medicine and Clinical Neurosciences, School of Medicine, Cardiff University, Cardiff, United Kingdom

**Keywords:** polygenic, PRS, alzheimer, PRSice, LDpred, PRS-CS, risk

## Abstract

Genome-wide association studies have identified nearly 40 genome-wide significant single nucleotide polymorphisms (SNPs) which are associated with Alzheimer's Disease (AD). Due to the polygenicity of AD, polygenic risk scores (PRS) have shown high potential for AD risk prediction. PRSs have been shown to successfully discriminate between AD cases and controls achieving a prediction accuracy of up to 84% based on area under the receiver operating curve. The prediction accuracy in AD is higher compared with other complex genetic disorders. PRS can be restricted to SNPs which reside in biologically relevant gene-sets; the predictive value of these gene-sets in the general population is not as high as genome-wide PRS, but they may play an important role to identify mechanisms of disease development and inform biological experiments. Multiple methods are available to derive PRSs, such as selecting SNPs based on statistical evidence of association with the disease or using prior evidence for SNP selection. All methods have advantages, but PRS produced using different methodologies are often not comparable, and results should be interpreted with care. Similarly, this is true when PRS is based on different background populations. With the exponential growth in development of digital electronic devices it is easy to calculate an individual's disease risk using public databases. A major limitation for the utility of PRSs is that the risk score is sample and method dependent. Therefore, replicability and interpretability of PRS is an important issue. PRS can be used to determine the probability of developing disease which incorporates information about disease risk in the general population or in a specific AD risk group. It is essential to consult with genetic counselors to ensure genetic risk is communicated appropriately.

## Introduction

Genome-wide association studies (GWAS) have identified over 40 genome-wide significant single nucleotide polymorphisms (SNPs) which are associated with Alzheimer's Disease (AD) (1–5). AD, similarly to other common genetic diseases and disorders, is now recognized to be polygenic (6–10). The polygenicity of AD leads to polygenic risk scores (PRS) being a successful approach in AD risk prediction. PRSs have been shown to discriminate between AD cases and controls achieving a prediction accuracy of 75–84% based on area under the receiver operating curve (AUC) in pathologically confirmed cases and controls (6, 9).

PRSs are advantageous for genetic prediction since genome-wide “*big data”* instigated by a large number of potentially contributing SNPs, are reduced into one variable which makes analysis much simpler by negating issues with overfitting and multiple testing penalties. PRSs account for the small effects of a large number of SNPs which still contribute to disease risk, successfully capturing the polygenicity of a disease. In AD, the PRS which includes all SNPs with *p* ≤ 0.5 shows the highest predictive ability, therefore, SNPs which show any association more than chance, may contribute to AD risk (6). PRS can be calculated at any point in an individual's life, so it is possible to assess disease risk prior to the onset of any disease or symptoms. This is particularly useful in late-onset diseases, or diseases which likely progress while an individual is asymptomatic, such as AD.

PRS has underlying requirements which result in a few limitations. Firstly it assumes independence between SNPs, therefore, SNPs in linkage disequilibrium (LD) are removed prior to analysis, resulting in loss of information. When PRS is derived for a dataset with individual genotype data, an independent summary statistic dataset is required to select the relevant SNPs. This is an increasing concern due to the amount of big data from large consortia which are produced using a meta-analysis of smaller datasets, where the independence is difficult to assess. Additionally, a *p*-value threshold is often used for SNP selection and this threshold may differ depending on genetic architecture of the disease and the power and quality of the data used for SNP selection (summary statistics). Without prior information, it is most common to test a number of arbitrary significance thresholds for SNP selection, and the threshold which optimizes prediction accuracy in a particular dataset is taken forward, this does however, incur a multiple testing penalty and is sample-specific. PRS assumes an additive model and interactions between SNPs are not taken into account.

The PRS distribution can be divided into cases and controls; the ideal scenario would show distinct separation between the two distributions, however, there is a great deal of overlap between the two, with the majority of individuals residing in the central part of the joint distribution. It is, therefore, not straightforward to use PRS to distinguish between cases and controls in general, but the distribution tails show better separation. Finally, because a large number of SNPs are incorporated into the score, it can be labor intensive to tease out which SNPs are driving the PRS, to further understand the biology of the disease.

## Methodology

In order to address the limitations highlighted in the introduction, there have been a number of methods developed to generate the PRS, these include PRSice, POLARIS, LDAK, LDpred, and PRS-CS. The original method using LD pruning and *p*-value thresholding will be referred to as PRS(P+T) in the remainder of the manuscript.

The traditional process, PRS(P+T), is to (1) remove SNPs in LD using intelligent pruning which retains SNPs with the strongest association with the disease, (2) choose SNPs which have a *p*-value below a defined threshold, and (3) compute the PRS on the remaining SNPs as a sum of the number of risk alleles, weighted by the SNP effect sizes (11). This method is very straightforward, but potentially removes a lot of information, and requires the specification of certain parameters, such as *p*-value threshold and LD pruning parameters (strength of LD, and the window size where the SNP × SNP LD is calculated). However, the simplicity of this method provides easily interpretable results, and a possibility to delve further into SNP profiles to see which SNPs are contributing to the PRS and how large this contribution is. The latter can be easily extracted from the summary statistics dataset.

PRSice (12, 13) is a software which implements the PRS(P+T) method. It tests a number of *p*-value thresholds in order to find the most appropriate for the particular dataset. One of the pitfalls is that the correction for testing at multiple *p*-values thresholds could be overlooked when association of the most appropriate PRS with the disease or trait is reported. In addition, since the *p*-value selection is based on the input data, the optimal *p*-value threshold may not be consistent across different datasets. Finally, the selected SNPs are dependent on LD structure of the input sample and even if the same parameters for PRS(P+T) and PRSice are specified, the resulting list of SNPs is very likely to be different for different input datasets. Nevertheless, PRSice is able to process very large datasets quickly, run regression models, adjust for covariates, plot results and restrict the PRS to pathways/gene-sets. The PRSice software is user-friendly, however, as with many software, it may take some investigation to understand all steps undertaken by the software, for instance, SNP removal due to ambiguity.

POLARIS (14) does not require LD pruning. It accounts for LD between SNPs across the full chromosome, and creates LD-adjusted genotypes, therefore enabling all SNPs to be included in the PRS. The contribution of each SNP is recalculated, usually downweighed accounting for the number of SNPs in LD and the strength of LD between them. The *p*-value threshold is also not required, however the pre-selection of SNPs based on *p*-values or any other criteria can be imposed prior to the analysis. This method is very computationally demanding since it requires the inversion of very large SNP × SNP LD matrices. The advent of cloud computing and cheaper hardware for computers may result in these methodologies becoming more accessible.

Similar to POLARIS, LDAK (15) was originally designed to adjust SNP effect sizes for LD, by reducing the contribution of SNPs in regions of high LD. Now, its primary function is to estimate SNP heritability and heritability enrichment in summary statistic data, whilst adjusting for local LD and allowing specification of the heritability model.

LDpred (16) and PRS-CS (17) are both Bayesian approaches which use a prior of SNP effect sizes and heritability captured by the regional LD structure. LDpred estimates the LD from datasets specified by the user. It can be either the same dataset which was used to generate the GWAS summary statistics or external (publicly available) data. In the current version of PRS-CS LD can only be estimated using the 1,000 Genomes data (18). While using publicly available QC-ed data (e.g., 1,000 genomes) can be useful to avoid additional pre-processing steps of individual genotype data provided to the PRS-software, researchers should be cautious about whether the sample used to estimate LD is representative of the summary statistic data, otherwise the adjustment for LD may be incorrect. This happens if the degree of concordance between Beta coefficients (effect sizes) for certain SNPs does not correspond to the strength and direction of LD between these SNPs. Both these methods attempt to adjust for local LD. LDpred uses a sliding window of size specified by the user, and PRS-CS divides the genome into 1,703 relatively independent chunks with around 500 SNPs. Both methods have been shown to predict well, with PRS-CS outperforming LDpred whilst maintaining computational efficiency, due to the use of continuous shrinkage priors. PRS-CS gives very similar results to PRS(P+T), whereas LDpred performs slightly worse (17). These methods have their value, but due to the complex nature of the methodologies, it is difficult to determine exactly which SNPs are most impacting the PRS.

**Figure 1:**
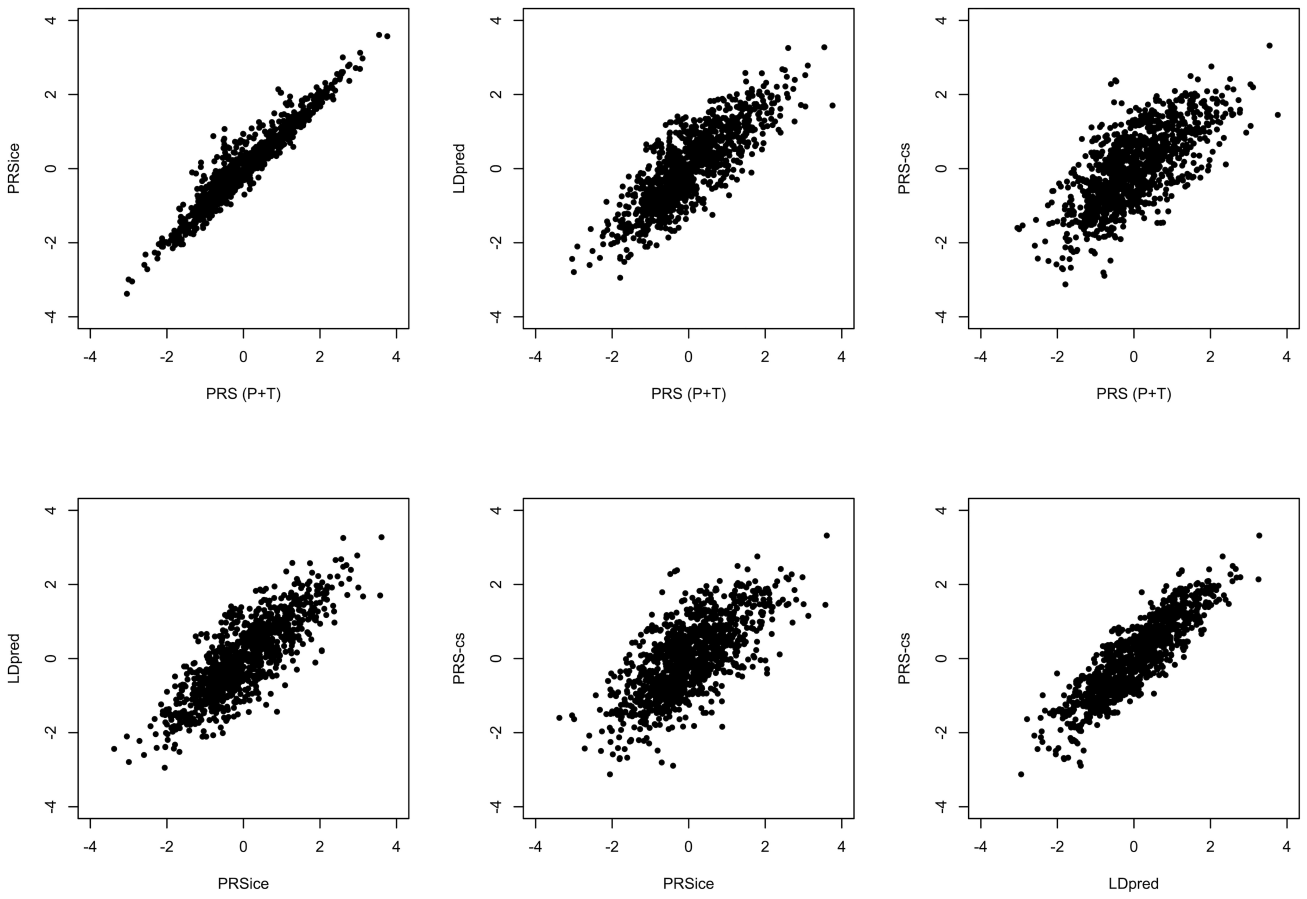
Comparison of PRS using Different Methodologies; PRS(P+T), PRSice, PRS-CS and LDpred. The PRS is computed for all individuals in HipSci (19) open access data merged with the 1,000 Genomes data (18), scores are weighted using AD GWAS (5) summary statistics. Only SNPs with *p* ≤ 0.5 are included and the *APOE* region (chr19: 44.4-46.5Mb) is excluded for all methods.

**Figure 2:**
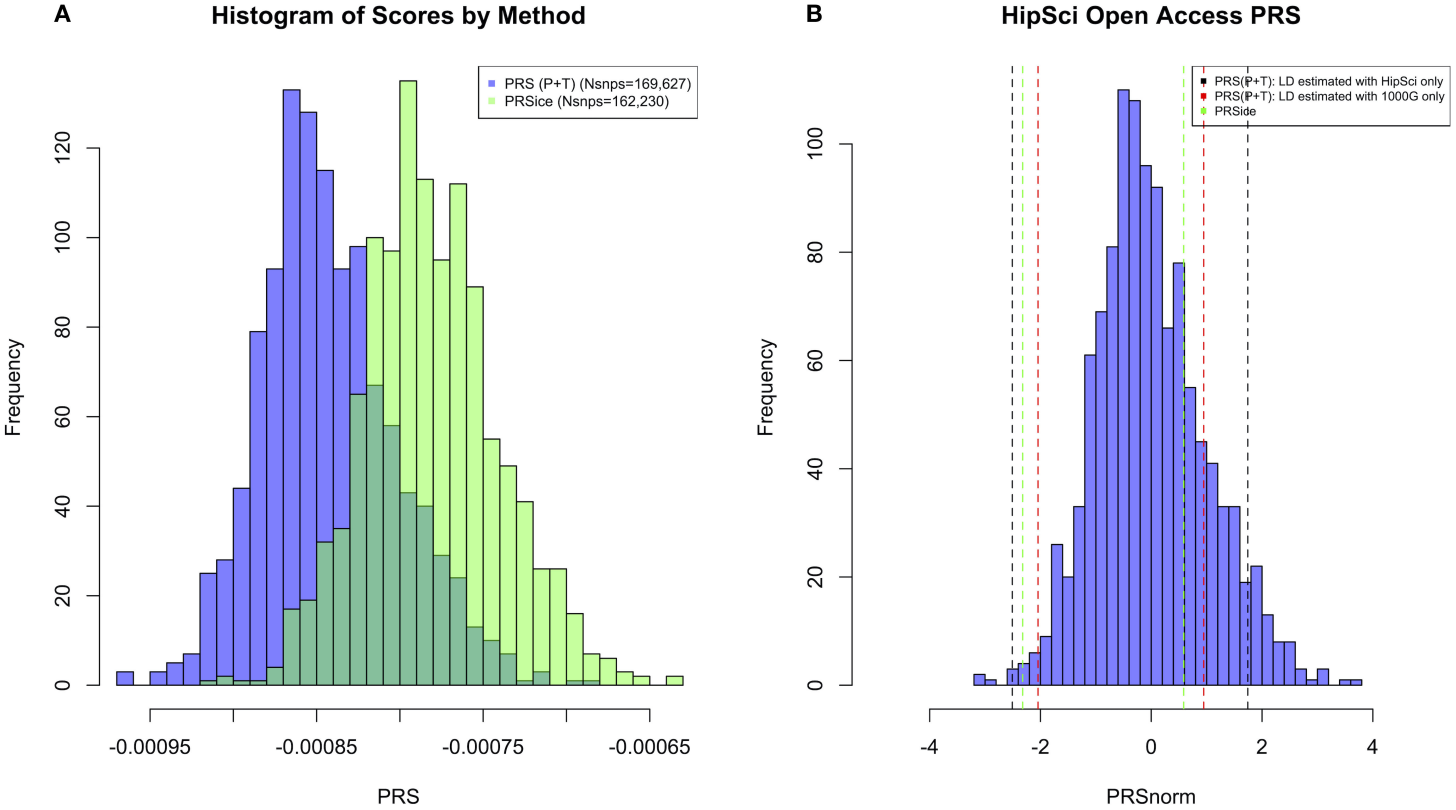
**(A)** Histogram of PRS Scores in HipSci (19) open access data + 1,000 Genomes data (18) for both PRS(P+T) and PRSice methods. Only SNPs with *p* ≤ 0.5 are included in the score, and a clumping threshold of 0.1 was used. **(B)** Histogram displaying PRS extremes using PRS(P+T) estimating LD in HipSci (19) data. Additional vertical lines display the PRS of the same two individuals when LD is estimated from a different sample, or PRS is computed using a different method.

All the methods presented compute PRS in a different manner. All of them will pick up a polygenic signal in a sample *en-masse*, however, when attempting to interpret and communicate the PRS value for a particular person, PRSs are difficult to compare and interpret. In order to demonstrate these discussion points in real data, we generated a PRS in the HipSci open access data (19) merged with the 1,000 genomes data (18), weighted with AD GWAS (5) summary statistics. Only SNPs with *p* ≤ 0.5 were included into the score, and the *APOE* region was excluded (chr19: 44.4-46.5Mb). The PRS was produced using PRS (P+T), PRSice, LDpred, and PRS-CS; for PRS (P+T) both HipSci and 1,000 genome data were used separately to estimate LD. Figure 1 shows scatterplots between the standardized PRS from PRS(P+T), PRSice, PRS-CS, and LDpred. As expected, PRS(P+T) and PRSice are the most correlated approaches. PRS(P+T) is least correlated with PRS-CS and LDpred. Figure 2A shows the unstandardised PRS distributions generated in the same sample, with the same SNPs. Due to additional filtering by PRSice, the number of SNPs in the score differs and the distributions show a systematic shift [this is more pronounced with other software (see Figure 1)]. An individual's PRS generated with a different software may differ dramatically [e.g., green and black vertical lines in the positive part of the PRS distribution in Figure 2B, indicating the same person's PRS calculated with PRS(P+T) and PRSice]. Figure 2B also demonstrates that PRS is sensitive to the sample used to estimate LD (compare black and red vertical lines).

Since the disease architectures defined by the SNP specific disease models are likely to be different, some areas of the genome should not be modeled within the PRS. For example, amyloid protein precursor (*APP*), presenilin 1 (*PSEN1*), and presenilin 2 (*PSEN2*) genes contain rare highly penetrant dominant mutations (20), which if modeled as a part of PRS will demonstrate very little contribution to the disease risk. Another example is the *APOE* gene. *APOE* has a very strong genetic effect on the risk of developing AD (21); many SNPs both within and around *APOE* are in high LD and show a strong statistical association with AD. Since *APOE* is so influential in AD, the greatest prediction accuracy of the case/control status is given when *APOE* is modeled separately to the PRS (6). The number of ε4 and ε2 alleles are modeled against AD, and an *APOE* weighted score using the effect sizes from these models as weights is produced. This is then included in the model with PRS, where the effect of *APOE* is excluded from the PRS by excluding all SNPs in the region (chr19:44.4-46.5Mb). Unfortunately, standard software do not have the ability to select disease specific regions which ought to be modeled separately or removed from the risk score. This is an important feature which PRS methods would benefit from if incorporated into the software.

For prediction in clinical settings, methods to date do not incorporate environmental factors which would be a beneficial addition in order to further explain more disease heritability and improve prediction. Current PRS methods allow us to capture SNP-based (narrow-sense) heritability. The incorporation of environmental factors, SNP × SNP and SNP × environment interactions, rare variants and SNP-specific disease models (e.g., dominant, recessive, etc.) into the risk score may enable the capture of broad-sense heritability (22, 23). In addition, when prediction accuracy is calculated, reported and communicated, the adjustment for relevant confounding factors, population stratification in particular, have to be considered. The software discussed do not explicitly provide these adjustments.

## Standardization of the PRS

Comparability of PRS across different populations and datas is one of the most important issues which requires addressing. PRSs computed in different populations are not necessarily comparable. For individuals from different ethnic backgrounds, the SNPs which are selected for the score may differ and LD structure between SNPs and SNP allele frequency will vary based on ethnicity. For example, in our in-house analyses, PRS values of ~10 have been observed for individuals with African ancestry when the remainder of the individuals in the sample have European ancestry. To date, the PRS approach has mostly been used in European populations, but even multiple PRSs from the same population may not be comparable and require harmonization before statistical standardization (24). A GWAS conducted in samples of European ancestry and used for PRS calculations in other populations, may still be predictive of the disease risk but accuracy is likely to be reduced, especially in samples of African ancestry (25).

In addition, other characteristics of a sample need to be carefully considered and accounted for. For example, since AD is an age-related disorder and disease prevalence varies depending on age, sample age may impact the PRS distribution and consequently, the prediction accuracy of the PRS. For example, AD prevalence in the general population is around 2% (26), in individuals who are aged 65+ prevalence is around 10% (27) and in those aged 85+ prevalence increases to 30% (28). It is also known that the effect of *APOE* is weaker in the age group 85+ (29). Therefore, PRSs should account for such factors before statistical standardization to ensure scores are comparable between samples and interpreted correctly. Since PRS is comprised of many risk variants of small effect, scores are Normally distributed but differences in factors (such as age and ancestry) will be reflected in the parameters of the PRS distribution [mean and standard deviation (SD)]. For easy interpretation PRS are often standardized *within* the study to make the mean=0 and SD=1. However, this does not make the scores comparable *between* studies, as the original (unstandardised) mean and SD may have been different due to the specifics of the sample (age, gender, ancestry, education etc.).

## PRS for Functional Studies

The identification of gene mutations that alter risk for a disease is an important route to understanding the disease mechanism. For common sporadic AD, the genetic risk is dispersed over a large number of variants and, with the exception of *APOE*, variants have small effects and most occur in non-coding regions where the functional variant/s are ambiguous. It is also clear that the genetics of common AD is underpinned by several components or pathways, which combine together to trigger disease. Pathway analysis of AD shows significant patterns of association implicating immunity, lipid processing, endocytosis, ubiquitination and more recently, Abeta and tau processing (5, 30). Cell type specific expression patterns have repeatedly associated the AD polygenic signal with microglia-specific gene expression patterns while recent single cell dissection of AD post-mortem tissue has found microglia dysfunction as a significant early event, all identifying microglia as a key AD cell type.

Most commonly, a genome-wide PRS is used across all available SNPs in the data, however, it is possible to restrict the SNPs to those within biologically relevant genes. Generally, these do not show as good prediction accuracy as the genome-wide PRS (31). Nevertheless, to improve prediction ability, the goal is to lower the number of SNPs whilst maintaining the heritability explained by the SNPs (23), thus reducing the signal-to-noise ratio. The hope is to make the risk score much more interpretable when it is comprised of the effect of biologically important SNPs. Identifying a small number of hub genes whose perturbation can capture the larger polygenicity will also be key for therapeutically targeting these networks.

PRS is often used to test the polygenicity and predictive ability of genetic data. By investigating individuals at the extremes of risk for a particular disease, PRS has other applications. For example, it is possible to create a PRS for iPSC lines, and identify and study cell lines which are at risk extremes. Since the biological experiments are quite expensive, selecting polygenic extremes can increase confidence in the cell line developing disease or remaining a control. In addition, PRS could be used to recruit individuals into clinical trials, by taking those most and least likely to develop a disease, such as AD, it is possible to increase the statistical power of your study.

The use of PRS to identify individuals at the extremes of risk is a promising approach, however, the interpretation of what it means to be in the “extremes” of a risk distribution should be taken with caution. For example, if the AD polygenic risk was computed for a group of people who happen to have very low risk for AD, when this risk is standardized (to give a distribution with a mean of 0 and standard deviation of 1), some individuals may look to be positive extremes, at high risk of developing AD, however, when compared to the general population, even the positive extremes are at lower risk compared to the rest of the population. Therefore, PRS alone should not be used to determine AD risk, but instead a probability of developing disease which incorporates information about the disease prevalence in the sample and in the general population. To minimize this effect, the PRS can be standardized against a population sample, this helps to identify extremes based on the general population. To do this, your data of interest has to be merged with a population sample with similar ancestry, such as the 1,000 Genomes data (18), and PRS is computed on the merged sample. The PRS is then standardized based on the mean and standard deviation of the PRS in the 1,000 Genomes sample. Once individuals at the extremes of risk are identified; the most influential SNPs which drive the PRS and differentiate between positive and negative extremes can be highlighted using a GWAS of SNPs in the score, for extreme individuals.

With the reducing cost of genetic testing, and the rise of companies offering this service to the public, the importance of genetic counseling is ever increasing. Much research has been done by clinicians and genetic counselors to guide how to communicate genetic risk (32). Individuals may prefer not to know their genetic risk of late-onset disorders such as AD, especially when there is no available intervention or treatment.

Genetic prediction based upon PRS alone is insufficient for precision medicine. An approach based upon combination of genetics, environmental factors, and disease biomarkers is necessary. To be able to use precision medicine, an understanding of the relationship between genotype and phenotype is required. This understanding would aid in targeting treatments and interventions, based on both the individual and disease characteristics (33, 34).

## Conclusions

PRS has the potential to be a very useful resource in complex genetic diseases to suggest diagnosis in the early phase of illness when patients present with very general and non-specific symptoms. Prediction by a genetic component alone can contribute to the risk prediction accuracy, however, it is unlikely to be a stand-alone predictor of a specific disease. When results are communicated to individuals, a careful explanation needs to be provided clearly separating a genetic test with a very high predictive value (e.g., rare fully penetrant mutations), and the PRS which alone has a limited predictive value. With the appearance of commercial companies which provide genetic data inexpensively to the general public, doctors might soon face the challenge of explaining PRS risk to individuals. The comparability of PRS values at the personal level, and therefore a unified approach to PRS generation and standardization will become more of an issue in the near future (if not already) in the digital health field. There are still a number of technical and methodological issues which need to be resolved, but as the field moves in the direction of PRS, these will likely be addressed and the utility of PRS in complex disorders will be substantial.

## Author Contributions

All authors listed have made a substantial, direct and intellectual contribution to the work, and approved it for publication.

## Conflict of Interest

The authors declare that the research was conducted in the absence of any commercial or financial relationships that could be construed as a potential conflict of interest.
